# The lived experiences of children living on the streets of Hillbrow

**DOI:** 10.4102/curationis.v38i1.1274

**Published:** 2015-05-22

**Authors:** Chris Myburgh, Aneesa Moolla, Marie Poggenpoel

**Affiliations:** 1 Department of Educational Psychology, University of Johannesburg, South Africa; 2 Department of Nursing Science, University of Johannesburg, South Africa

## Abstract

**Background:** The effects of daily abuse and hardship on the streets lead to poor mental health in children living on the streets, resulting in them choosing ineffective and self-destructive coping strategies that impact their physical health and overall sense of wellbeing. The facilitation of the mental health of children living on the streets who are subjected to daily threats to their survival is thus crucial.

**Objectives:** The aim of this research was to explore and describe the lived experiences of children living on the streets of Hillbrow, Johannesburg.

**Method:** The research design was qualitative, exploratory, descriptive and contextual. A purposive sample was selected through a temporary shelter in Johannesburg, Gauteng, South Africa and consisted of 14 male children living on the streets. Data were collected using drawings, in-depth phenomenological interviews and field notes. The central interview opening statement was: ‘Tell me about your life on the street’.

**Results:** The results obtained indicated that children living on the streets are threatened, exploited and exposed to physical, sexual and emotional abuse on a daily basis by the community, the authorities and other street dwellers. This leads to feelings of sadness, fear, anxiety, misery, despair, hopelessness, helplessness and suicide ideation, which in turn lead to drug abuse and criminal activities. In contrast, positive feelings of sympathy for other children living on the streets emerged and these children also displayed perseverance, resilience and a striving for autonomy.

**Conclusion:** Street life exposes children to a variety of experiences, both positive and negative. A striving after autonomy is clearly depicted by these children, who are able to tap into a range of responses, both on- and off-street.

## Introduction

### Background

As a global community, people are becoming more aware of the necessity of addressing the needs of each and every child at risk. Of particular interest in this study are children living on the streets – a visible manifestation of a fragmented socioeconomic and political society. The phenomenon of children living on the streets is experienced both here in South Africa and internationally as a psychosocial–educational challenge (Le Roux 2001; Moolla 2012:1). Over time, it has come to be recognised that it is not enough to simply place children living on the streets in institutions, but that it would be more meaningful and helpful to understand who these children are, as individuals (Tudorić-Ghemo 2005:xii). Knowing how they function on an emotional and psychological level, as well as gaining an understanding of their beliefs, values and dreams, have profound implications for any interventions aimed at improving the quality of their lives. Understanding the needs of children living on the streets in greater depth might provide them with the opportunity to be included in decision-making processes that are pertinent to their future as contributing members of society. According to McAlpine et al. (2010:27) the ‘“street” experience is highly individual and contextual’. Not nearly enough research has been done to learn about the depth and diversity of the street experience directly from the perspectives of street children themselves and what they think might help (Panter-Brick 2002:156).

According to Rydberg (2006:1–4), many children living on the streets suffer as they are constantly exposed to drug abuse, sickness, violence and hunger. Other studies have also found that there is a high incidence of mental health challenges, such as addiction to glue sniffing, depression and aggression resulting from the circumstances of living on the streets (Gracey 2002; Moolla 2007:65–78; Uys & Middleton 2014:725). Much of this has been attributed to the abusive, dysfunctional and neglected home environments that these children come from and is generally exacerbated by the lifestyle that they lead on the streets. Children living on the streets around the world tend to suffer from the consequences of their daily battle for survival in the street context. Such a harsh environment affects all areas of functioning of children living on the streets. Furthermore, the self-esteem of children living on the streets is low, not only because of what they do but because of treatment they receive from the public. They are seldom treated with respect or offered assistance, which tends to instil in them a negative vision of life and the future (Jamison 2008:17). Various other studies have confirmed that children living on the streets are indeed not criminals nor deficient of character; rather, many are running away from their lives which are rooted in multi-problem households rife with poverty, hunger, alcohol abuse, family violence, family dissolution and in the breakdown of traditional supportive community structures (Cockburn 2004:46; Gracey 2002:6; McAlpine et al. 2010:32; Panter-Brick 2002:152; Tudorić-Ghemo 2005:61).

Schimmel (2006:227) further noted that these experiences of stigma can cause children living on the streets to internalise the negative perceptions that members of the public have of them. They also tend to exhibit antisocial and self-destructive behaviour through joining gangs and/or engaging in crime. In addition, the context of their intra-personal communication is impeded to such an extent that children living on the streets’ behaviour becomes less adaptive and more defensive, affecting their interpersonal skills as well as social roles. These children living on the streets experience any removal from their context – for instance, being forced into homes or schools – as distressing and as a confusion or disturbance of their identities (Schimmel 2006:227).

According to De Vries (2009:24), children living on the streets thus tend to develop networks of friendship with people who have similar lifestyles. The insecurity of their lives and their daily struggle to find food, work and shelter, avoid confrontation with law enforcement and the public, then makes them dependent on other children living on the streets which has another vast array of influences. This was stated by Kidd (2003:250), who noted that coping strategies most mentioned were hanging out with friends and taking drugs or alcohol. These strategies were described as relaxing and as a social activity by which one can bond with friends on the street. However, they also found having friends counteracted feelings of hopelessness, loneliness and worthlessness and can thus be noted as an important factor in the survival of children living on the streets (Kidd 2003:245–253).

According to Van Blerk (2006):

[*t*]he diversity of children’s and youths on-street experiences – manifest in the ways they use public spaces to survive, show preferences and display personalities – suggest multiple possibilities in the use of agency [*or autonomy*] at the margins. (p. 47)

Children are often the most affected by adverse circumstances because of their relative immaturity and their lack of social power (Boyden & Mann 2005:3). According to Gigengack (2008:13), the individual life courses of children living on the streets tend to follow the street life cycle, namely, if children survive living on the streets, they are likely to become youths, young adults, veterans and seniors of streets and institutes; and the main exit from street life is death.

In previous research (Moolla, Myburgh & Poggenpoel 2008:597), it was found that children living on the streets are more vulnerable to impaired mental health than any other group of children. Children living on the streets around the world tend to suffer the consequences of their daily battle for survival in the street context. Such a harsh environment affects all areas of functioning of children living on the streets.

### Problem statement

Children living on the streets of Johannesburg are confronted with challenges mostly related to survival. These challenges are on intra- as well as interpersonal levels. Children living on the streets seemingly experience harsh realities of constantly being on the alert. However, very little research is available concerning the lived experiences of children living on the streets of Hillbrow, Johannesburg. Thus, against the above background, the following research question was formulated: ‘What are the lived experiences of children living on the streets of Johannesburg?’

#### Aim of the study

In accordance with this research question, the aim of this research was to explore and describe the lived experiences of children living on the streets of Hillbrow, Johannesburg.

## Research design

A qualitative design was chosen for this study to explore and describe how children living on the streets make sense of their lived-experiences on the street both as individuals and also their shared meanings with other children living on the streets.

### Population and sample

The population for this study consisted of adolescents living on the street who visited a temporary shelter in Hillbrow frequently. The gatekeepers in this shelter were social workers who looked after the wellbeing of the children living on the streets. Purposive sampling (Burns & Grove 2011:355) was used, where the children living on the streets who would most likely render information-rich data were selected. The sampling criteria were that the children living on the streets had to be between 8 and 17 years of age, were living on the street for at least 1 year, visited the temporary street children shelter frequently and were able to communicate in English or Zulu.

### Data collection

The eligible participants were invited to draw a picture depicting their life on the street. These drawings done by the children living on the streets were utilised as the point of departure for in-depth phenomenological interviews conducted with the participants (Moolla 2012:60–61). According to Patterson and Hayne (2009:124), ‘young children reported more information when asked to draw and tell about a past emotional event than children only asked to tell’. The interviews were based on the drawings done by the children living on the streets and they were asked the question: ‘Tell me about your life on the street’.

Follow-up questions to facilitate the interview (Creswell 2007:125) were asked to assist the participants to elaborate on a particular point and to gain an understanding of their experiences as children living on the streets. Interviews were conducted until data saturation was obtained (Creswell 2008:257–258). According to Kvale (1996:102) and Holloway and Wheeler (2010:146–147), data saturation occurs where further interviews add little new knowledge. Field notes were made of observations (Holloway & Wheeler 2010:117) during the interviews.

### Data treatment

All the interviews were audio-taped, transcribed and analysed using Tesch’s open coding (Creswell 2008:154–155; 2012:243–244). This meant that all of the transcripts and field notes were read to get a sense of the whole of the participants’ experiences of living on the streets. During this process, units of meaning, namely, units relevant to the participants’ experiences of living on the streets, were identified. These units of meaning were then labelled. After the units of meaning were identified and described they are translated into themes (Patton 2002:453). The themes were then supported by direct quotations from the participants (Creswell 2007:125, 209). An independent coder was used during the data analysis stage of the research. A consensus discussion between the independent coder and the researcher was held before and after data analysis was conducted. A literature control was then done to verify the results.

## Results

The sample consisted of 14 male children, living on the streets for more than 3 years, who were 8 to 17 years of age.

Five major themes were identified as reflecting the daily lived experiences of children living on the streets (Moolla 2012:135–136). These themes are:

- Theme 1: Experiences of being a street child is a way of life.
- Theme 2: Children living on the streets experience that they are exposed to risks and threats of life in all contexts of their daily living on the street.
- Theme 3: Children living on the streets exhibit various emotional responses to their daily lived experiences.
- Theme 4: Children living on the streets develop various coping strategies against the harsh environment.
- Theme 5: Children living on the streets show resilience by striving for autonomy.

These themes are now discussed, with relevant quotations from the interviews.

### Theme 1

Being a street child is a way of life. It is the only reality with no alternative lifestyle in mind. Children living on the streets choose to live an autonomous and nomadic life on the street rather than being neglected in the family-of-origin context. A variety of family-of-origin factors contribute to the child leaving home. These factors include: being physically assaulted by caregivers who often abuse alcohol; being blamed for no reason; poverty; abandonment and rejection by parents and other family members; and becoming orphans.

Even though living on the street is also associated with a lack or avoidance of formal schooling, many children living on the streets in this study were actually literate but had not completed their school education.

These children experience autonomy and self-reliance that stems from living on the streets. One child living on the street said:

‘It is better to be on the street … because I can get food … because I am begging … then I can get money for food … it is better this way … It is better on the street because I can make my own money by begging … and buy what I want … food … and it makes me happy to have money.’ (Participant 5, 8 years old)

They take responsibility for their lives and view living on the street as being better than being neglected by family. A participant said:

‘So this place was not too good for us because like my father he like to drink you see … stuff like this … *ya*… he like … he was … he used to leave us alone and he go and he come back like late *ya*… And he beat us.’ (Participant 3, 13 years old)

To attend formal schooling is seen as a stumbling block as they are ‘acting’ older than their actual age. They do not want to be treated as children anymore. As one said:

‘But when you go to school … they are treating you like a small boy. Yes … when I was staying with my grandmother…but they are treating you badly in school. So I left … they are treating us like small children.’ (Participant 2, 11 years old)

### Theme 2

Children living on the streets are frequently exposed to physical assault, threats and verbal abuse by other groups of children living on the streets; the general public and the police. Older, bigger and more experienced children living on the streets often exert dominance over younger children.

Police harassment and assault is a serious hazard to these children. This highlights the powerless position in society of children living on the streets. A participant explained:

‘Also, sometimes the police they are coming to take us and they are saying … hey boys come here … they just take you somewhere but you can’t trust them because the police they used to beat us.’ (Participant 7, 12 years old)

Children living on the streets are exploited by various individuals who offer them money in exchange for criminal or sexual favours. As one participant said:

‘Then those people they come, they say how much? Then maybe sometimes I say twenty rand … and then the other one they come they say … I don’t have twenty, I just only got fifteen rand. I say okay … I do it because I want money you see. That’s why I leave it because I didn’t feel nice because sometimes you get the one that got lot big dick you see.’ (Participant 10, 15 years old)

Children living on the streets are also constantly exposed to harsh environmental conditions without shelter. The inability to maintain personal hygiene is another significant discomfort of living on the street and the outcome of this is the onset of dermatological conditions. The following participant said:

‘Then we sleep in the pipe there. *Ya*, in the pipe. Ehh, the way you are staying … when it becomes too cold … then you have the other thing … what can I say it’s like a small animal you see. It starts to bite you because you don’t bath yourself and this animal goes inside your body … inside your skin.’ (Participant 8, 16 years old)

Begging as a source of income is frequently supplemented with stealing to obtain money which indicates that children living on the streets engage in this criminal behaviour to support their drug use, not so much to meet their needs for food. Drug addiction is an important factor that prevents children living on the streets from escaping their context and to choose in favour of a better life. A participant said:

‘When they are smoking the glue, they feeling good like they can do anything, like they are in a cartoon. They are feeling like everything is not real … so they are feeling like they are in a cartoon, they feel brave. They feel like they can do whatever they want to. It can be that they can kill people and it will feel like not real.’ (Participant 13, 17 years old)

### Theme 3

Children living on the streets exhibit various emotional responses to their daily life experiences, which impact on them in many different ways.

#### Experiences of negative emotions

The children living on the streets in this study experience a deep sadness, a longing for physical safety, maternal nurturing and care; and also a sense of identity and personal worth. Being dehumanised and subjected to the coercive powers and physical abuse by police and other children living on the streets, abandonment by significant others, seeking medical help; and being exploited for sexual favours also leads to fear, anxiety, misery and survival despair in children living on the streets. Participants said:

‘I run away every time because I am scared. They want our money, they are making us scared. We give them the money because they are scaring us.’ (Participant 1, 10 years old)

‘Yes, we are not feeling well. We are feeling very bad, too much bullying. I am feeling very terrible. Sometimes they getting mad, I feel bad. They are also forcing me to smoke. I am looking for some shelter all the time because I am scared.’ (Participant 14, 15 years old)

‘They are treating me like a slave. Whenever they buy something, then they want you to hold it, then they give you nothing, they shout and chase you.’ (Participant 12, 16 years old)

#### Experiences of positive emotions

Participants showed deep care and concern for the plight of other children living on the streets. Children living on the streets also feel upset when they witness other children living on the streets – even the ‘bad boys’ – getting hurt. One said:

‘I always run away if I see the people is getting hurt because I don’t want to see that. I feel very bad and upset …’ (Participant 9, 12 years old)

Living on the street sensitises children living on the streets to the plight of other children living on the streets. It is a form of survivorship to be actively involved in helping other children living on the streets. Another participant stated:

‘You must help these children … they smoke glue.’ (Participant 6, 14 years old)

#### Experiences of suicide ideation

It was also noted that there was an indication of suicide ideation – a subtle expression of wishing to die and escape from the difficult life on the street by being reunited with a significant family member. A participant said:

‘I want to follow my father then … but he is gone too far …’ (Participant 12, 16 years old)

This indication of suicide ideation is likely a result of a sense of abandonment, social isolation and lack of social support in the lives of these children living on the streets. Another participant said:

‘It was very bad and I had nobody to help me … I wanted to die …’ (Participant 13, 17 years old)

### Theme 4

Children living on the streets display various defense mechanisms against the harsh environment, which include sublimation, suppression and displacement (Sadock, Sadock & Ruiz 2015:162).

#### Sublimation

‘The gratification of an impulse whose goal is retained but whose aim is changed from a socially objectionable one to a socially valued one.’ (Sadock, Sadock & Ruiz 2015:162)

Children living on the streets’ frequent relocations expose them to significant risks and their lives are thus characterised by recurrent running away and hiding, either from their homes of origin or from personal risks, dangers and threats on the street. A participant explained:

‘Then I run away whenever I see the police car … I run away every time because I am scared and when you are small then you get bullied by the big boys, so I run away and hide.’ (Participant 13, 17 years old)

In addition, life on the street also meets children living on the streets’ primary need for social interactions and for belonging to a significant group. One advantage of friendships is social networking with other children living on the streets that may provide access to resources that addresses their primary needs and personal protection against the risks of physical assault. Another participant stated:

‘I was staying with another friend of mine who was older than them. They were scared of him you see. If they try anything like that I just tell him … he go beat them. He used to be a friend of mine … a good friend of mine … he used to help me a lot.’ (Participant 10, 15 years old)

#### Suppression

‘A “conscious” or semiconscious decision to postpone attention to a conscious impulse or conflict.’ (Sadock et al. 2015:162)

Drug use allows children living on the streets to temporarily escape into a fantasy world where everything is good and where the longing for being in meaningful relationships is inhibited. Children living on the streets have to be innovative and devise ways to meet their primary food needs since they are responsible for their own wellbeing:

Begging from the general public is also often supplemented with stealing to obtain money. Participants shared the following:

‘Those friends if they want food they go out to like a Shoprite they ask food. Others go to Pick & Pay and ask there, others go to the dustbin. They do all those stuff … something like that.’ (Participant 8, 16 years old)

‘If you didn’t smoke the drugs you feel lonely … you feel like we are sick you see. You feel cold even if there is a fire you feel cold … cold. It’s the Taiwan … It’s a heroin … glue …’ (Participant 12, 16 years old)

Drug-using children use their money to buy drugs, whilst they meet their need for food by searching refuse bins:

‘If you didn’t smoke the drugs you feel lonely … you feel like you are sick. You feel cold even if there is a fire … It’s the Taiwan … It’s heroin … glue …’ (Participant 12, 16 years old)

‘… [*A*]ctually everyone who stays on the street goes to beg or to steal and comes back and smoke … that’s the only thing we do when we are staying on the street …’ (Participant 3, 13 years old)

#### Displacement

‘A purposeful, unconscious shifting of affective investment from one object to another in the interest of solving a conflict.’ (Sadock et al. 2015:162)

Citizens from other African countries are viewed as being the primary offenders of organised crime groups that recruit children living on the streets. It is difficult to verify the factual correctness of this statement. However, it may be seen as a form of xenophobia. Participants shared:

‘The Nigerians … they are buying the stolen things from everyone.’ (Participant 10, 15 years old)

‘Too many people you see … Somali and Nigerians … [*take street children to shoplift for them*] … It’s the people who live around the town … the Nigerians … most of them are Nigerians … *Ya*… you can only just buy it [*drugs*] from them …’ (Participant 12, 16 years old)

‘[*T*]hey always taking money from those other people … they are from Mozambique.’ (Participant 7, 12 years old)

### Theme 5

Children living on the streets striving to be free often display defiance and perseverance against the coercive power of other groups. This is a striving for personal autonomy and perhaps also a striving to be a ‘good’ person who is not involved in criminal behaviour, despite everyday survival challenges. It also seems as though the participants in this study were not actively forced to leave home, but rather decided autonomously to leave and live on the streets. This possibly explains their assertive behaviour and sense of autonomy in their current context. Life on the street is regarded as a better lifestyle and choice than living in a difficult and abusive family context.

These children living on the streets showed a determination to leave and to escape their difficult and abusive family context. One participant said:

‘My stepfather was shouting and screaming then beating me, so I left. My mother and my stepfather … they come there to fetch me. Then I run away again and then he is beating me up again for running away. I feel happy … I am happy now … I am far away now from home.’ (Participant 3, 13 years old)

Children living on the streets in this study understood that certain actions have clear negative consequences that should be avoided by doing the ‘good’ thing. Refusing a verbal demand by the ‘*esilunde*’ – older delinquent children living on the streets – also requires courage because of the consequences, namely, assault. Some of the participants in this study chose, radically, to part from a certain group of friends and a life of prostitution to start a meaningful life in a formal shelter. These children stated:

‘I am not smoking anything … not dagga and not cigarettes. I was always staying separate because the others. They were smoking … they are also forcing me to smoke but I said I do not want to smoke. I never smoked … I don’t want to start now.’ (Participant 6, 14 years old)

‘I destroy all those things to come here to Twilight to do these beads. So I want my life to be changed … I want to do something that’s going to be nice for me.’ (Participant 14, 15 years old)

## Ethical considerations

Ethical clearance for this research (clearance number 254/14/09/2009) was obtained from the Research Ethics Committee of a Faculty of Education at this university. Ethical measures, such as autonomy, non-maleficence, beneficence and justice, were adhered to during the research process (Dhai & McQuoid-Mason 2011:14–16). Autonomy were adhered to by discussing the research with the gatekeepers of the temporary shelter of street children, including the management team and social worker. The social worker explained the research to the street children and assured that their participation was voluntary and that they might withdraw at any time without any consequences to them. All participants assented to participate after the research method was explained to them. No incentives were offered to the street children, although fruit juice and snacks were available for all participants. Those participants who needed psychological support after the interviews were debriefed by the social worker at the temporary shelter. The drawing and interviews were anonymous and confidential. The interviews were taped with the permission of the participants. The data were kept under lock and key in a cupboard in the researcher’s office. Only the researchers had access to the data. The data will be destroyed two years after publication of the research.

## Trustworthiness

In order to ensure the validity and reliability of this research, use was made of Guba’s (Lincoln & Guba 1985:290) model of trustworthiness. Techniques to achieve credibility included prolonged engagement in the field and varied field experiences. Transferability was achieved by dense description of results, description of sample realisation and purposive sampling. Techniques to achieve dependability included triangulation of data research methods, dense description of research methods and code–recode procedures. Finally, confirmability was achieved with the use of reflexivity, an audit trail and triangulation.

## Discussion

Other authors support the finding that street children experience being on the street as a way of life. McAlpine et al. (2010:32) confirmed that children living on the streets are not deviants; rather, many are escaping from their lives which are rooted in problematic households rife with poverty, hunger, alcohol abuse, family violence and family dissolution, as well as in the breakdown of community networks (Malindi & Theron 2010). A study by Owoaje, Adebiyi and Asuzu (2009:13) has also brought forth a peculiar type of children living on the streets who still attend school but are likely to eventually abandon school because of a lack of adult supervision and the influence of street influence on them. Many children living on the streets, particularly boys, reported being ‘drawn to the relative freedom and independence of the streets, and valued this very highly once on the streets’ (Kudrati, Plummer & Yousif 2008:439). According to Finkelstein (2005:42), establishing street networks is not only for survival purposes. Forming street networks helps children living on the streets not only learn the ropes, but also allows them to experience the rewards of friendship ties and develop resilience (Ungar 2007; 2011).

### Children experience living on the streets exposing them to risks and threats of life

More than 50% of children living on the streets report being threatened regularly with weapons, attacked physically and abused, both verbally and sexually, on the streets in order to commit crimes to get money, drugs and alcohol. If they are prostituting from the street, they are likely to be addicted, involved with a pimp and become more susceptible to arrest – all factors that quickly exacerbate their homelessness. ‘As their addiction begins to take over, they make less money and the money they have goes for drugs’ (Boyer 2008:21).

Children living on the streets exhibit various emotional responses to their daily lived experiences, including the experience of negative emotions and suicidal ideation. Research indicates that children living on the streets are more vulnerable to impaired psychological health than any other group of children (Tudorić-Ghemo 2005:66). Further studies indicate that psychological responses to abuse, such as anxiety, fear, anger, denial, self-hypnosis, disassociation and self-mutilation, are common (Celik & Baybuga 2009:21; Valente 2005:10–11). Children living on the streets learn early to distrust the assertation that the world is a safe place, resulting in a loss of a sense of faith and a sense of hopelessness. In addition, the instability of life on the streets only exacerbates the child’s lack of trust and a healthy sense of identity and overall personal trustworthiness becomes negatively distorted (Motala & Smith 2003:63).

In a study conducted by Schimmel (2006:212), children living on the streets experience high levels of stress and of physical and sexual abuse and psychological trauma as a result of living on the street. They suffer from psychological pathologies, such as depression and suicidal behaviour, at substantially higher rates than children who live at home or in alternative permanent accommodation. Other studies have also concluded that street youths present with serious emotional and behavioural disturbances, suicide or suicidal ideation, anxiety and depression, low self-esteem, withdrawal, feelings of hopelessness, inferiority and despondency (Richter & Van der Walt 2003:11).

Children living on the streets develop various coping strategies against the harsh environment, including sublimation, suppression and displacement. According to Van Blerk (2005:14), children living on the streets also ‘participate in strategies to help them elude the police such as hiding or leaving town for a few weeks’. According to Finkelstein (2005:39), newcomers on the street seek out other children that can help them. Earlier hardships lead to these children becoming involved with more experienced children who offer them support and the tools they need to survive. Harper (2003:32) states that in recent years, as more attention has been drawn to the existence of children living on the streets, many health and welfare organisations, social workers and religious organisations have responded by providing intervention strategies and programmes to address the basic needs of children living on the streets.

According to a study by Tudorić-Ghemo (2005:100), glue, marijuana and alcohol were most common amongst the youth and all had used some form of substance or drug. Peers, who are the main sources of socialisation and support for street children, are often highly influential in introducing street kids to behaviours such as substance abuse, stealing, prostitution and other illicit modes of street life (Finkelstein 2005:40). Substance abuse use often provides children living on the streets with an escape from the harsh realities of street life. Many feel that substance use gives them a way to remain emotionally on the streets. It keeps them ‘out there’, so to speak (Finkelstein 2005:92).

Valji (2003:1) is of opinion that:

[*a*]n examination of [*the xenophobia*] phenomenon and its manifestation reveals that ‘the foreigner’ has become a site for the violent convergence of a host of unresolved social tensions. The difficulties of transition, socio-economic frustrations, a legacy of racial division, and an inherited culture of violence are just some of the factors contributing to violent xenophobia in South Africa today.

Children living on the streets show resilience by striving for autonomy. Children are often treated like outlaws by local authorities once they are on the streets. The migration to the street may in fact suggest an act of personal resilience. These children believe that ‘they have a better chance to further their own lives and livelihood in positive ways by leaving home prematurely’ (McAlpine et al. 2010:30). According to Cockburn (2004:46), children living on the streets find support and camaraderie amongst their peers on the street, but they are looking beyond what the peer group has to offer. Running away from intolerable circumstances can be evidence of extraordinarily adaptive behaviour.

## Limitations of the study

A once-off study may not be sufficient enough to obtain an in-depth understanding of how it is for children to live on the streets.

## Recommendations

More shelters should be made available for children living on the streets. A professional nurse also needs to be part of the team so that the children can receive health and mental health promotion information as well as support from adults. The support from adults should be to assist children living on the street to manage stress and to cope with their traumatic experiences of living on the street. The adults should also explore with these children possible other ways of living than living on the street.

## Conclusion

Street life exposes children to a variety of experiences which can disrupt their world-view and image of reality. Interactions with the general public, the authorities and other children living on the streets, range from positive to negative. These ambiguous experiences lead to feelings of confusion, despair, helplessness and suicide ideation in children living on the streets.

A striving for autonomy is clearly depicted as children living on the streets strive to be free from abusive guardians and from the coercive power of other children living on the streets groups. It includes acts of defiance and perseverance against the coercive power of these individuals and groups. Ultimately, this indicates an attitude of hope and being a survivor rather than a victim of circumstances. The children living on the streets in this research strive to lead a morally good life which involves the ability to distinguish between good and bad actions. There is clearly the ability to appreciate cause and effect linked to criminal behaviour and negative outcomes.

Children living on the streets make a choice to engage in processes of mobility, which impacts on their actual identity. This mobility affords them a number of opportunities that enhance their survival strategies and resilience on the street. They are able to tap into a range of resources on-street and off-street locations at different times, enabled by their fluid identities.
